# Maternal mixed UPD3 and a homozygous PLXNA1 c.2497G>C variant in a fetus with severe anomalies

**DOI:** 10.3389/fmed.2025.1712148

**Published:** 2026-01-23

**Authors:** Yanchou Ye, Xiaonan Wang, Yunxia He, Haofeng Ning, Zhechao Zhang, Fangchao Tao, Zhangxiang Zou, Qun Fang, Zheng Chen, Xiaohui Tian, Xiulan Hao

**Affiliations:** 1 Prenatal Diagnostic Center, Department of Obstetrics and Gynecology, The Seventh Affiliated Hospital, Sun Yat-sen University, Shenzhen, China; 2 Department of Obstetrics and Gynecology, The Seventh Affiliated Hospital, Sun Yat-sen University, Shenzhen, China

**Keywords:** NIPT, trio-CMA, trio-WGS, mixed UPD3, PLXNA1

## Abstract

**Background:**

Non-invasive prenatal testing (NIPT) is widely used for screening common fetal aneuploidies such as trisomy 21 (T21), trisomy 18 (T18), and trisomy 13 (T13). However, its utility in detecting trisomy 3 (T3) has been rarely reported. Furthermore, uniparental disomy (UPD) involving chromosome 3 is a rare genetic condition with potential phenotypic consequences.

**Methods:**

NIPT indicated a high risk for fetal T3. This finding was further investigated using copy number variation (CNV) analysis via trio-based chromosomal microarray analysis (trio-CMA). Subsequent trio-based whole-genome sequencing (trio-WGS) identified a homozygous variant in PLXNA1 associated with a putative autosomal recessive disorder in the fetus. The detected variant was validated by Sanger sequencing in the parents.

**Results:**

NIPT revealed a fetal *Z*-score (27.22) for T3. Trio-CMA ruled out T3 but confirmed mixed maternal UPD3. Trio-WGS identified a homozygous PLXNA1 variant (NM_032242.3:c.2497G>C, p.Ala833Pro) in the fetus, inherited from the heterozygous mother. The observed severe fetal phenotype was partial consistent with the molecular findings of mixed UPD3 and the homozygous PLXNA1 variant, indicating that this variant may represent a potential pathogenic cause.

**Conclusions:**

While NIPT can signal a high risk for rare aneuploidies, definitive diagnosis requires invasive prenatal testing. Discrepancies between NIPT and fetal tissue analyses may arise from confined placental mosaicism (CPM). We propose a model in which nondisjunction of chromosome 3 during germ cell formation led to trisomy, followed by a postzygotic self-correction event, resulting in mixed maternal UPD3 and increased risk of autosomal recessive disorders.

## 1 Introduction

T3 is one of the rarest autosomal trisomies detected via NIPT (1). While the likelihood of true fetal aneuploidy is low, such cases are associated with an elevated risk for adverse obstetric outcomes and UPD (2).

UPD occurs when both copies of a chromosome are inherited from one parent. Mechanisms underlying UPD include trisomy rescue, monosomy rescue, gametic complementation, and somatic recombination. UPD is further categorized into heterodisomy (hetUPD), which involves the inheritance of both non-identical homologous chromosomes from one parent, and isodisomy (isoUPD), which involves the inheritance of two identical copies of the same chromosome. UPD can also be classified as maternal (matUPD) or paternal (patUPD) based on the parental origin (3). UPD can lead to disease by unmasking autosomal recessive disorders or disrupting the expression of imprinted genes. Specific phenotypic outcomes have been well-documented in matUPD for chromosomes 7, 11, 14, 15, and 20, and in patUPD for chromosomes 6, 11, 14, 15, and 20 (4–6). While UPD has been reported for all chromosomes except the Y chromosome, it is most frequently observed on chromosomes 1, 4, 16, 21, 22, and X (7). However, although chromosome 3 does not harbor well-characterized imprinted genes, isoUPD3 increases the risk of autosomal recessive disorders.

Here, we report the first case of a fetus with matUPD3 carrying a homozygous PLXNA1 variant. The fetal phenotype, which included early-onset fetal growth restriction (FGR), abnormal cerebellar development, ventricular septal defect, persistent truncus arteriosus, abnormal venous duct development, and left congenital diaphragmatic hernia, was observed alongside both the matUPD3 and the homozygous PLXNA1 genotype. These findings highlight the clinical value of comprehensive trio-based analysis in diagnosis for delineating potential genotype-phenotype correlations, thereby informing genetic counseling and pregnancy management.

## 2 Materials and Methods

### 2.1 Subjects

This study involved a 34-year-old primigravida (G1P0) and her 35-year-old husband, both with no significant family genetic history (data collected in 2024), who presented at the Prenatal Diagnostic Center of the Seventh Affiliated Hospital of Sun Yat-sen University for routine screening. Written informed consent was obtained from all participants, and the study protocol was approved by the Hospital Ethics Committee (No. KY-2025-382-01).

### 2.2 Methods

#### 2.2.1 NIPT

Maternal peripheral blood (5 ml) was collected in Streck Cell-Free DNA BCT^®^ blood collection tubes (Streck, La Vista, NE, United States) at a gestational age of 12 weeks. Cell-free DNA (cfDNA) was extracted from 200 μL of maternal plasma. Library preparation included end repair, adapter ligation, and PCR. Amplified double-stranded DNA was thermally denatured into single-stranded DNA, cyclized, and converted into DNA nanoballs (DNBs). The DNBs were loaded onto sequencing chips and sequenced on the MGISEQ-2000 platform (BGI, Shenzhen, China) at the 0.1X average coverage. Raw reads were aligned to the genome version GRCh37/hg19 using BWA. Uniquely mapped reads were selected using SAM tags, and PCR duplicates were removed. *Z*-scores were calculated to detect fetal chromosomal aneuploidies and CNVs.

#### 2.2.2 Ultrasound examination

**Figure 1:**
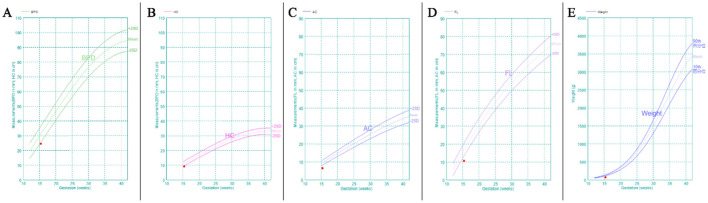
Fetal biometry at 15 weeks demonstrating severe growth restriction. GA (LMP) 15w3d, GA (AUA) 13w5d. **(A)** BPD value 2.46 cm, *Z* score −2.34. **(B)** HC value 9.20 cm, *Z* score −2.46. **(C)** AC value 6.48 cm, *Z* score −4.71. **(D)** FL value 1.06 cm, *Z* score −3.57. **(E)** EFW 73 g ± 11 g, *Z* score −10.78. GA, gestational weeks; LMP, last menstrual period; AUA, average ultrasound age; BPD, biparietal diameter; HC, head circumference; AC, abdominal circumference; FL, femur length.

**Figure 2:**
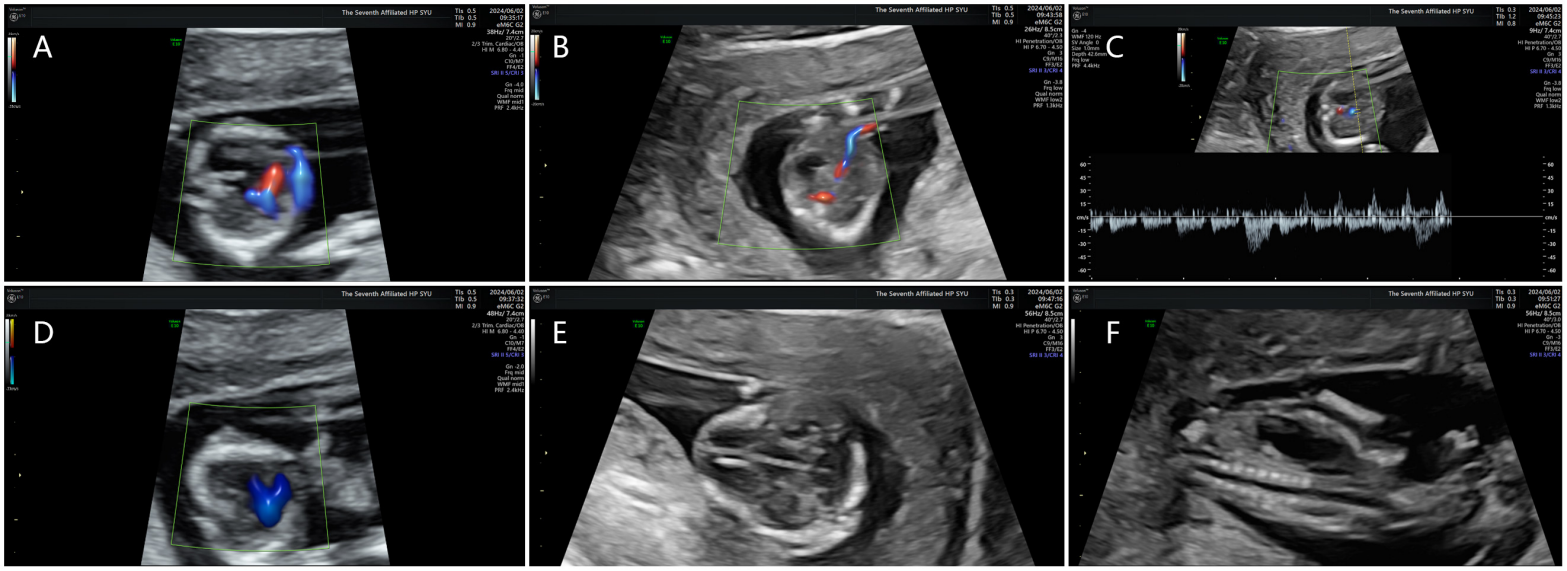
Diverse fetal anomalies identified on prenatal ultrasound. **(A)** Ventricular deficiency. **(B)** Abnormal development of venous ducts. **(C)** Intravenous catheterization spectrum reverse a-wave. **(D)** Common trunk of great arteries. **(E)** abnormal cerebellar development. **(F)** left congenital diaphragmatic hernia.

A subsequent comprehensive ultrasound examination at 15 weeks of gestation revealed severe FGR (Figure 1) in conjunction with multiple severe congenital structural anomalies (Figure 2), detailed as follows:

Cardiac Anomalies: persistent truncus arteriosus was suspected on fetal echocardiography, characterized by a single great artery overriding a large VSD (~1.6 mm), rightward cardiac displacement, and mild tricuspid regurgitation.
Cerebellar and CNS abnormalities: severe cerebellar hypoplasia was suspected based on the marked hypoplasia with indistinct hemispheric contours and an obliterated posterior fossa cistern.
Diaphragmatic Hernia: a left-sided congenital diaphragmatic hernia was suspected, given the visualization of the stomach in the left hemithorax with resulting mediastinal shift and compression of the left lung.

Given these findings, the pregnancy was terminated after genetic counseling. The parents declined a fetal postmortem examination, which limited further phenotypic characterization.

#### 2.2.3 Trio-CMA

Approximately 10 ml of amniotic fluid was collected and centrifuged at 3000 rpm for 10 min to obtain the cell pellet. Genomic DNA (gDNA) for CMA was then extracted from the pellet using the QIAGEN DNA Mini Kit (Cat. 51306; QIAGEN, Hilden, Germany) according to the manufacturer's instructions. A total of 250 ng of extracted gDNA was amplified, labeled, and hybridized to the Affymetrix CytoScan 750K array following the manufacturer's protocol. Raw data were analyzed in Chromosome Analysis Suite (ChAS) and annotated to GRCh37. CNV calls required ≥50 contiguous probes and a minimum size of ≥100 kb. Runs of homozygosity (ROH) were reported and interpreted in clinical context; large ROH (>10 Mb), particularly when restricted to imprinting chromosomes (6, 7, 11, 14, 15, 20), prompted evaluation for possible UPD. Detected CNVs were interpreted with reference to the scientific literature and public databases.

#### 2.2.4 Trio-WGS

Trio-WGS was performed on gDNA extracted from the fetal tissue and on parental blood gDNA. Each gDNA (300 ng) was enzymatically fragmented, and the resulting fragments underwent magnetic bead-based size selection (200–300 bp). After end repair and adapter ligation, the library was purified and PCR-amplified. Quality-controlled libraries were circularized and digested to generate DNBs. The DNBs were pooled and sequenced on the DNBSEQ-T7 platform (PE100+10). Reads were processed with SOAPnuke (8) and aligned to GRCh37/hg19 using BWA; GATK was used for SNV/indel calling (9), followed by annotation and filtering against population and disease databases. Variant interpretation followed American College of Medical Genetics and Genomics (ACMG) guidelines (10), and exome-derived single nucleotide polymorphism (SNP)/ROH information was leveraged to assess segmental UPD.

#### 2.2.5 Sanger sequencing

To validate the identified variant, primers were designed to flank the target regions. PCR amplification was performed, and the amplicons were subjected to Sanger Sequencing. The obtained sequences were compared against the reference sequence of the PLXNA1 gene (NM_032242.3:c.2497G>C) to confirm the findings from WGS.

#### 2.2.6 3D modeling of protein structure

The 3D structures of the wild-type and mutant proteins were predicted using AlphaFold3, as described by Josh Abramson et al. (11). Five independent models were generated for each variant. The model with the highest predicted Template Modeling (pTM) score was selected for subsequent analysis. The selected structures were visualized and analyzed using PyMOL (v2.5.6) to assess the structural impact of the variant. Final figures were annotated and rendered in Adobe Illustrator 2021 CC. We emphasize that this computational analysis serves only as a predictive tool to generate a testable structural hypothesis, not as functional validation.

## 3 Results

### 3.1 NIPT

**Figure 3:**
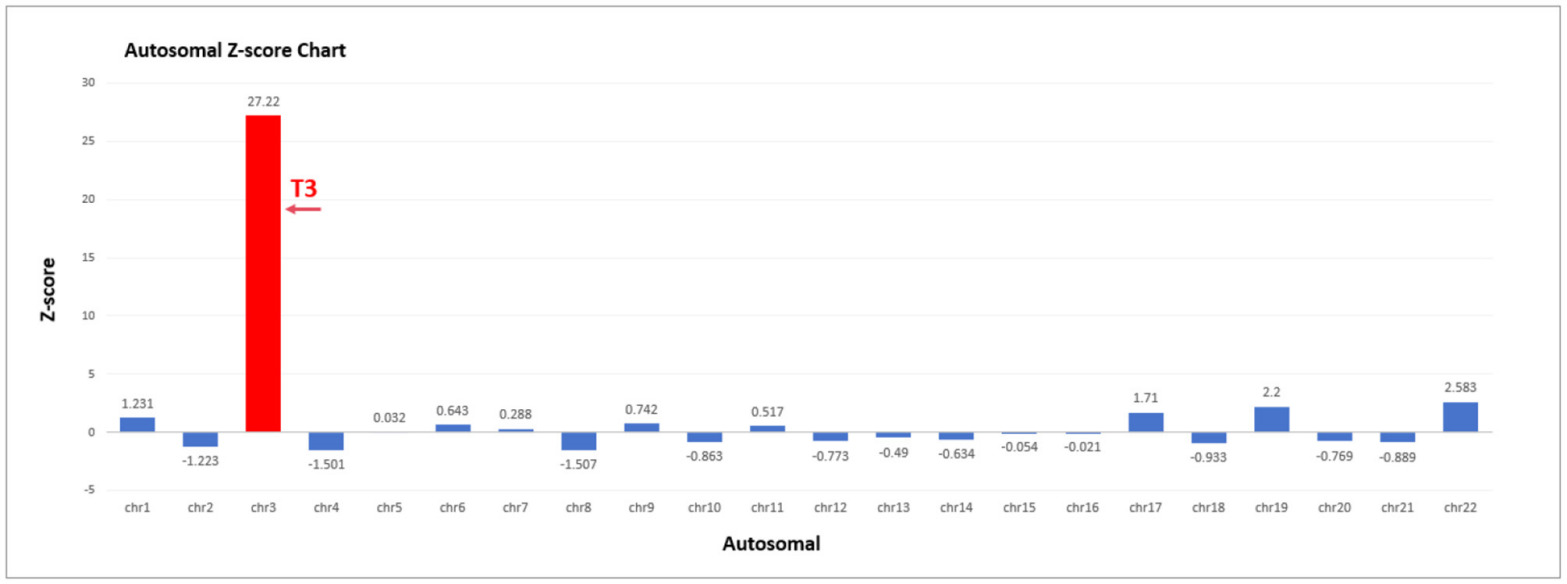
Autosomal *Z*-score chart in NIPT. Fetal fraction (FF): 16.12%. *Z*-score for T3 was 27.22.

The fetal NIPT result indicated a high risk for T3. Several quality control metrics were used to evaluate the sequencing data. The fetal DNA fraction was 16.12%. The Q30 score was 93.89%, indicating the proportion of bases with a quality score exceeding 90%. The number of unique reads was 7.29 million, and the *Z*-score for T3 was 27.22 (Figure 3).

### 3.2 Trio-CMA

**Figure 4:**
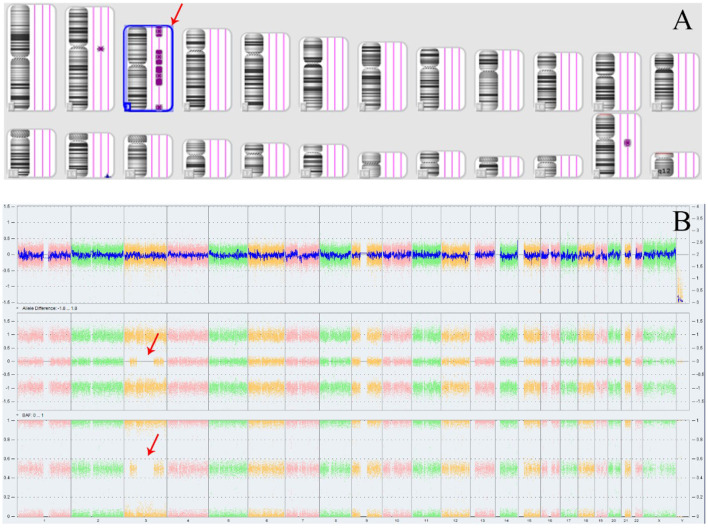
ROH results in Affymetrix CytoScan 750K CMA. **(A)** ChAS revealed segmental ROH across the entire chromosome (purple rectangle, red arrow). **(B)** A whole chromosome view clearly shows the copy neutral ROH on chromosome 3 in the fetus (red arrow). ROH, runs of homozygosity.

**Figure 5:**
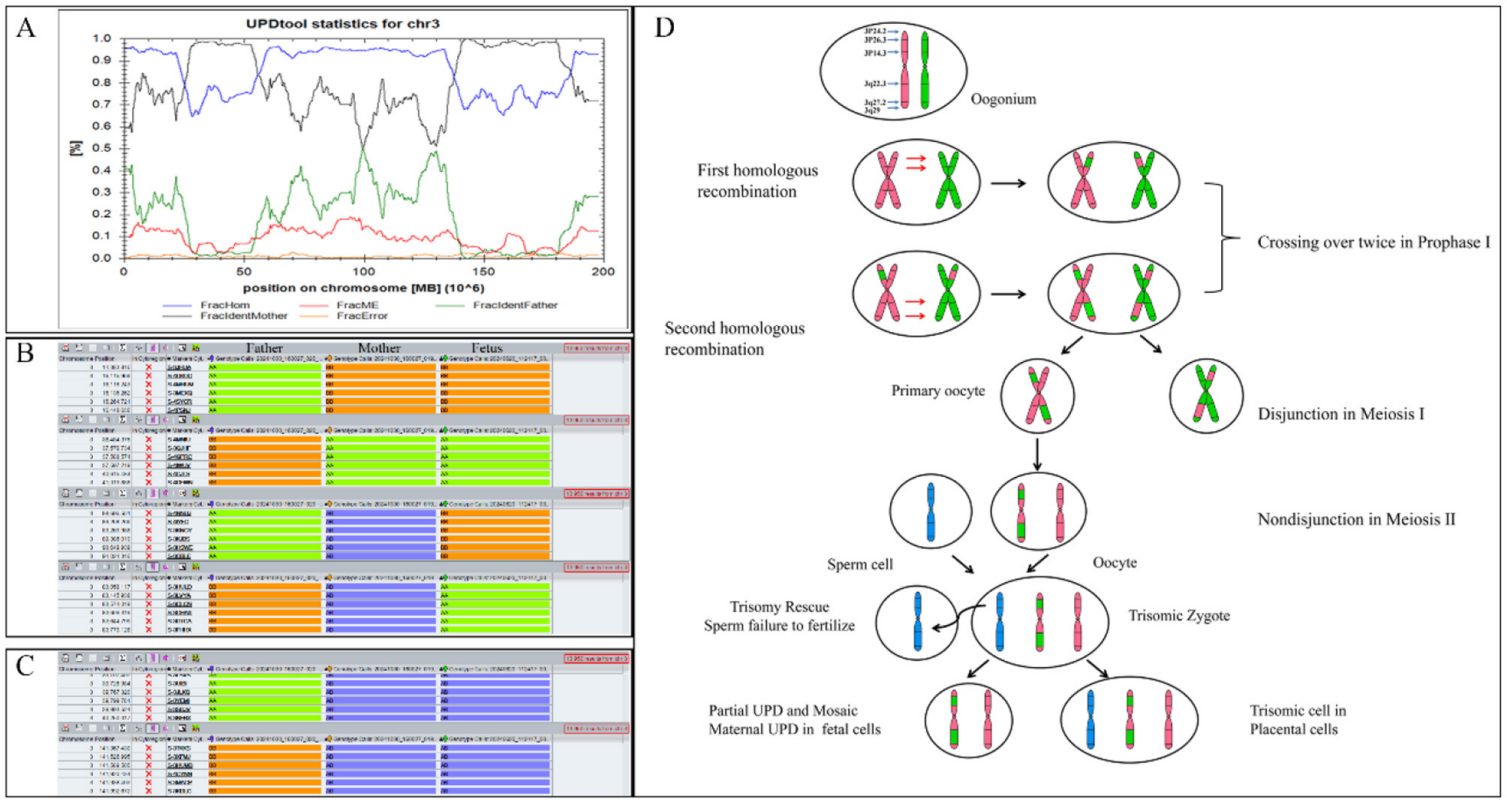
Analysis of segmental maternal UPD3 and its underlying mechanism. **(A)** Classification of UPD using the UPDtool showed the fetus was segmental maternal isoUPD. FracHom (blue line) is the fraction of homozygous SNPs, FracME (red line) is the fraction of mendelian error SNPs, FracldentFather (green line) is the fraction of SNPs where the genotype is identical to the father, FracldentMother (black line) is the fraction of SNPs where the genotype is identical to the mother, and FracError (yellow line) is the fraction of errors. **(B)** ChAS software directly indicates that the segmental isoUPD originated from her mother after comparing the genotyping results between the fetus and her parents, father's alleles were AA, mother‘s alleles were BB, fetal alleles were BB, or father's alleles were BB, mother‘s alleles were AA, fetal alleles were AA, or father's alleles were AA, mother‘s alleles were AB, fetal alleles were BB, or father's alleles were BB, mother‘s alleles were AB, fetal alleles were AA (blue arrow). **(C)** ChAS software directly indicates that the hetUPD originated from her mother after comparing the genotyping results between the fetus and her parents, father's alleles were AA, mother‘s alleles were AB, fetal alleles were AB, or father's alleles were BB, mother‘s alleles were AB, fetal alleles were AB (red arrow). **(D)** The postulated mechanism for the formation of mixed UPD. Segmental isoUPD was caused by nondisjunction in meiosis I after two crossing overs had happened, resulting in sections of isodisomy and heterodisomy on the UPD chromosome 3. isoUPD, isodisomy uniparental; hetUPD, heterodisomy uniparental.

The Affymetrix CytoScan 750K CMA did not identify any pathogenic or likely pathogenic CNVs in the fetus. However, four ROH segments were detected on chromosome 3 (Figure 4; Table 1). SNP analysis of the trio-CMA revealed complete maternal UPD3 with both heterodisomic and isodisomic regions in the fetus, as shown by Chromosome Analysis Suite (ChAS) and UPD-Tool statistics (Figures 5A–C). These findings suggest the presence of eight breaks leading to two crossover events on chromosome 3 during maternal meiosis I (Figure 5D).

**Table 1:** ROH segments identified on chromosome 3 by CMA.

**ID**	**Microarray nomenclature**	**Type**	**Cytoband start**	**Cytoband end**	**Length**	**Classification**
1	arr[GRCh37]3p26.3p24.2(73,603_24,898,233)x2	ROH	p26.3	p24.2	24.8 Mb	VUS
2	arr[GRCh37]3p14.3p11.1(55,593,238_90,485,635)x2	ROH	P14.3	P11.1	34.8 Mb	VUS
3	arr[GRCh37]3q11.1q22.3(93,558,926_138,054,521)x2	ROH	q11.1	q22.3	44.4 Mb	VUS
4	arr[GRCh37]3q27.2q29(184,561,541_197,791,601)x2	ROH	q27.2	q29	13.2 Mb	VUS

ROH, runs of homozygosity; Mb, million bases; VUS, variant of unknown significance.

### 3.3 Trio-WGS

WGS revealed matUPD of chromosome 3, involving both isodisomy and heterodisomy regions in the fetus. The isodisomic regions included four segments of ROH segments: 22.5 Mb at 3p26.3–p24.3 (GRCh37:3:1–22,500,000), 33.4 Mb at 3p14.3–p11.1 (GRCh37:3:55,100,000–88,500,000), 45 Mb at 3p11.1–q22.2 (GRCh37:3:90,600,000–135,600,000), and 8.7 Mb at 3q27.1–q29 (GRCh37:3:184,100,000–192,800,000). Within these regions, a homozygous PLXNA1 variant (NM_032242.3:c.2497G>C, p.Ala833Pro) was identified. This variant was classified as a variant of uncertain significance (VUS) according to the ACMG guidelines, with PM2 (absent from controls), PM3 (homozygous due to UPD, confirming in trans status), and PP2 (missense variant in PLXNA1; the gene is highly intolerant to missense variation as indicated by a gnomAD missense *Z*-score of 3.45, and missense variants are a known disease mechanism for this gene). Its pathogenicity classification may change in the future as more data accumulate.

### 3.4 Sanger sequencing

**Figure 6:**
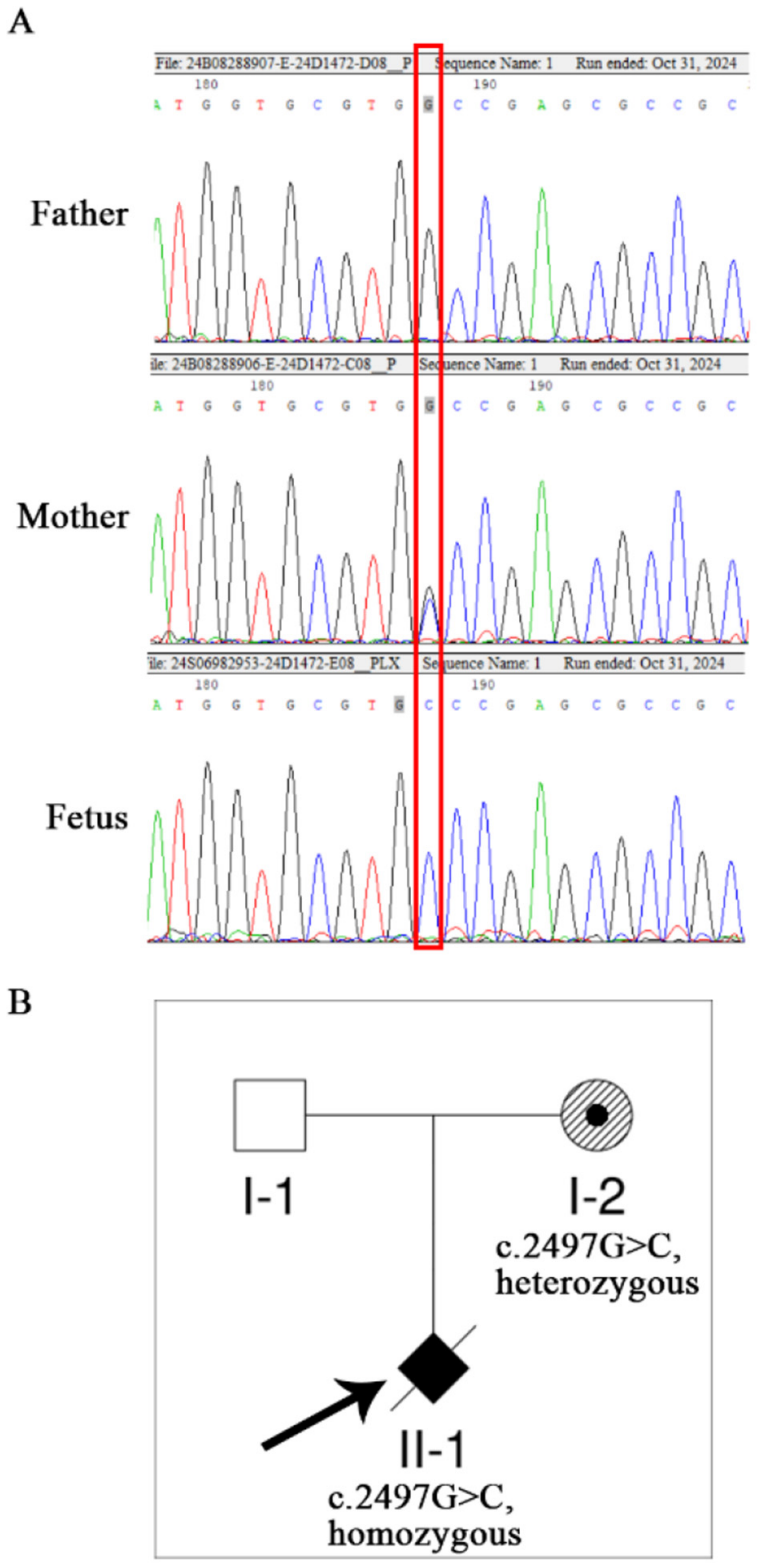
Sanger sequencing results of the PLXNA1 c.2497G>C variant in the pedigree. **(A)** Sanger sequencing chromatogram of PLXNA1, showing heterozygous c.2497G>C variant in the mother, homozygous c.2497G>C variant in the fetus, while wild type in father (Red frame). **(B)** The pedigree of the family with PLXNA1: c.2497G>C variant.

Sanger sequencing confirmed that the fetus was homozygous for the c.2497G>C (p.Ala833Pro) variant in the PLXNA1 gene. The mother was heterozygous for this variant, while it was not detected in the father. The corresponding family pedigree is provided (Figure 6).

### 3.5 3D modeling of protein structure

**Figure 7:**
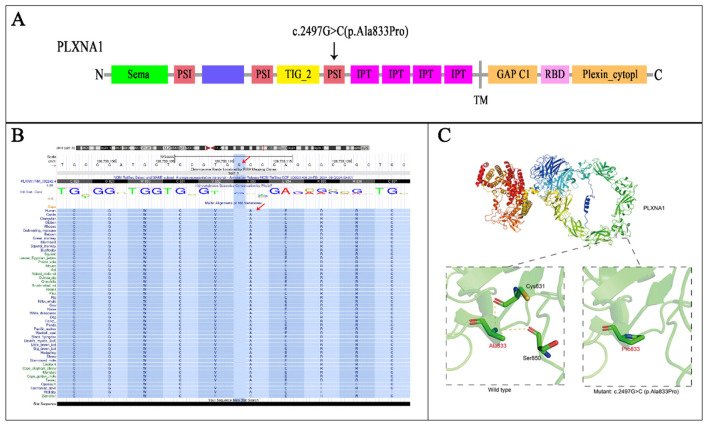
Structural and functional impact of the PLXNA1 p.Ala833Pro variant. **(A)** Schematic protein domain structure adapted from St. Clair et al. and https://www.deciphergenomics.org/. The biallelic variants with PLXNA1: c.2497G>C (p.Ala833Pro) is on the PSI domain. PSI, plexin, semaphorin, integrin domain; IPT, Ig-like, plexin, transcription factors domain; TM, transmembrane; GAP C1/2, split Ras-GTPase-activating protein domains; RBD, Rho family GTPase-binding domain. **(B)** A sequence alignment highlights the conservation of PLXNA1: c.2497G>C (p.Ala833Pro) across mammal with highly developed nervous system, sense organs, brain. **(C)** 3D protein structure prediction shows the wild type of PLXNA1, the Ala833 site forms hydrogen bonds with Cys831 and Ser850, thereby stabilizing the local structure of the protein. However, after mutated to Pro833, due to the special self-forming mechanism of proline, all the original interactions disappeared, which may reduce the stability of the protein, and make the protein precipitate and degrade.

In the wild-type PLXNA1 protein, the Ala833 residue forms hydrogen bonds with Cys831 and Ser850, which stabilizes the local structure. Upon substitution with proline (Pro833), however, the unique cyclic conformation and inherent rigidity of the residue disrupt these native interactions. This structural perturbation is predicted to reduce protein stability, potentially leading to misfolding, aggregation, and degradation (Figure 7).

### 3.6 . Literature review

A systematic literature search was performed in PubMed (https://pubmed.ncbi.nlm.nih.gov) using the search terms “UPD3” and “uniparental disomy chromosome 3.” Twelve relevant cases were identified, from which the following data were extracted: sex, prenatal and postnatal phenotypes, placental findings, UPD size, UPD type, karyotype, and additional genetic findings (Table 2).

**Table 2:** Clinical and molecular findings from published cases of UPD3.

**References**	**Published year**	**Age**	**Mat/Pat**	**Mat age**	**Pat age**	**Size of UPD3**	**Type**	**Gene**	**Variation type**	**Band**	**Disease**	**Other genetic findings**
Schollen et al. (13)	2005	4Y	Mat	–	–	3q21.3-3qter	Segmental UPD	ALG3: p.R266C (c.796C > T)	Homozygous	3q27.1	Congenital disorder of glycosylation type Id (CDG-Id)	*de novo* mutation
Hoffman et al. (14)	2007	23M	Mat	–	–	Chr3	Isodisomy	GLUT2:C1213T	Homozygous	3q26.2	Fanconi Bickel syndrome (FBS)	ABCC8: T1252C heterozygous mutation
Fassihi et al. (15)	2006	1Y	Mat	34	35	Chr3	Isodisomy	COL7A1:345insG	Homozygous	3p21.31	Hallopeau–Siemens recessive dystrophic epidermolysis bullosa (HS-RDEB)	46, XY
Srebniak et al. (36)	2008	12 weeks of gestate+on	Mat	36	–	Chr3	Isodisomy	NO	NO		IUGR, microcephatus	Mat UPD (3), der (3) pat
King et al. (16)	2014	1.5Y	Mat	–	–	Chr3	Isodisomy	GLB1:c.1038G > C(p.Lys346Asn)	Homozygous	3p22.3	GM1 gangliosidosis	–
Tahara et al. (17)	2020	1Y	Mat	–	–	Chr3	Isodisomy	LIPH:c.736T > A (p.C246S)	Homozygous	3q27.2	Woolly hair/hypotrichosis	–
Kopp et al. (18)	2021	18M	Mat	–	–	Chr3	Isodisomy	ABHD5:c.700C > T, p.(Arg234^*^)	Homozygous	3p22.1	Chanarin–Dorfman syndrome	*de novo* mutation
Xiao et al. (22)	2006	42Y	Paternal	19	26	Chr3	Isodisomy	NO	NO	NO	NO	46, XY
Matejas et al. (19)	2011	20D	Paternal	36	41	Chr3	isodisomy	LAMB2:c.1405+1G>A	Homozygosity	3p21.31	Pierson syndrome	46, XY
Myers et al. (20)	2017	10Y	Paternal	–	–	Chr3	mosaic UPD	GLB1:c.75+2dupT	mosaic paternal UPD, estimated at ~45% of cells in blood	3p22.3	GM1 gangliosidosis	–
Andolfo et al. (21)	2020	21D	Paternal	–	–	Chr3	Segmental UPD	SLC25A38:c.832C>T(p.Arg278^*^)	Homozygous	3p22.1	Sideroblastic anemias	–
Bu et al. (23)	2021	1.5Y	Paternal	46	59	Chr3	Isodisomy	NO	NO	NO	No physical abnormalities	46, XX

–, No mention; NO, not found.

## 4 Discussion

Previous estimates of UPD prevalence, ranging from 1 in 3,500 to 1 in 5,000, were derived from extrapolations based on clinically apparent UPD cases, which failed to account for chromosomal variability or UPD associated with healthy phenotypes. More recent data suggest that UPD is approximately twice as prevalent in the general population, with an estimated rate of 1 in 2,000 births (12). Regarding UPD specifically involving UPD3, a total of 12 cases have been reported, comprising 9 cases of complete UPD3, 2 cases of segmental UPD3, and 1 case of mosaic UPD3. Based on parental origin, 7 cases were maternal and 5 were paternal. Among the 7 maternal UPD3 cases with abnormal phenotypes, 6 were due to homozygous pathogenic variants of autosomal recessive disorders, and 1 exhibited an abnormal karyotype. The disorders associated with these cases included congenital disorder of glycosylation type Id (ALG3) (13), Fanconi–Bickel syndrome (GLUT2) (14), Hallopeau–Siemens recessive dystrophic epidermolysis bullosa (COL7A1) (15), GM1 gangliosidosis (GLB1) (16), woolly hair/hypotrichosis (LIPH) (17), and Chanarin–Dorfman syndrome (ABHD5) (18).

In the 5 reported cases of patUPD, 3 were linked to single-gene disorders, while 2 exhibited no apparent phenotypic abnormalities. The phenotypes associated with paternal UPD3 included Pierson syndrome (LAMB2) (19), GM1 gangliosidosis (GLB1) (20), and sideroblastic anemia (SLC25A38) (21). Notably, 2 cases of paternal UPD3 involving the entire chromosome were described without evident disease phenotypes. Xiao (22) reported a 42-year-old male who was identified through whole-genome linkage analysis and exhibited no overt phenotypic abnormalities. Bu (23) described a fetus with normal prenatal ultrasound findings; follow-up at 1.5 years revealed no significant growth or developmental abnormalities. Regarding the potential for imprinting on chromosome 3, although bioinformatics predictions have identified two maternally expressed genes (ALDH1L1 and ZIC2) and one paternally expressed gene (HES1), no functional imprinted regions or definitive imprinted genes have been documented. All reported UPD3 cases with abnormal phenotypes have resulted from homozygous variants in genes associated with autosomal recessive disorders.

In the present study, trio-CMA and trio-WGS identified large ROH segments encompassing the entire maternal chromosome 3, demonstrating a case of mixed maternal UPD3. We hypothesized that this mixed UPD3 resulted from two crossover events during maternal meiosis I, followed by a non-disjunction event. This meiotic error led to a combination of isodisomy and heterodisomy on chromosome 3. Following fertilization, the zygote was likely trisomic for chromosome 3. Trisomy rescue likely represents the mechanism by which this aneuploid zygote reverted to euploidy (24). Critically, trisomy rescue can result in a condition known as CPM, where the trisomic cell line is eliminated from the fetal lineage but persists in the placenta. During the blastocyst stage, the extra chromosome is lost in embryonic progenitor cells via mitotic division, while trisomy 3 persists in placental progenitor cells, resulting in a trisomy placenta (25). Given that circulating cell-free DNA (cfDNA) in maternal blood primarily originates from placental trophoblastic cells (26, 27), this CPM provides the most plausible explanation for the initial NIPT result indicating trisomy 3 in this case, rather than technical errors, low-quality samples, or negligence.

Given that maternal UPD3 can unmask recessive alleles within the ROH segments, we analyzed the trio-WGS data for potential pathogenic homozygous variants. Trio-WGS revealed the simultaneous detection of homozygous c.2497G>C (p.Ala833Pro) variant in the PLXNA1 gene (GRCh37:3:126733111). This variant falls within the maternal ROH segment spanning 3p11.1q22.2 (90,600,000–135,600,000). The mother was confirmed to be a heterozygous carrier. The variant, located in the plexin-semaphorin-integrin (PSI) domain, has not been previously reported in major public databases (GnomAD, ClinVar, HGMD, ExAC). *In silico* predictions for this variant were inconsistent: SIFT predicted it as deleterious, and it showed evolutionary conservation (GERP score 3.39), whereas some other algorithms suggested a polymorphic nature. A 3D modeling of protein structure indicated a potential impact on protein function. According to the ACMG/AMP guidelines, the variant was classified as VUS (10, 28). Thus, while the variant is a suspected contributing factor to the fetal phenotype, it cannot be definitively rated as likely pathogenic or pathogenic at this stage.

The fetal clinical features included FGR, abnormalities of the nervous system and vasculature, partially overlapping with the spectrum of Dworschak–Punetha neurodevelopmental syndrome, a novel neurodevelopmental disorder with developmental delay, brain and eye anomalies associated with PLXNA1 variants. The PLXNA1 gene encodes plexin A1, a transmembrane protein highly expressed in the developing nervous system. Plexins form complexes with Neuropilin-1 (NRP1) and function as receptors for class 3 semaphorins, key molecules in axonal guidance (29, 30). Their cytoplasmic domain contains segments homologous to GTPase-activating proteins (GAPs), forming a functional GAP domain (31), underscoring their crucial role in developmental signaling, particularly in the nervous and vascular systems.

Clinically, phenotypes linked to PLXNA1 mutations are variable, encompassing neurological, ocular, and cutaneous manifestations, global developmental delay, brain imaging abnormalities, and dysmorphic ventricles. To date, rare biallelic and monoallelic *de novo* PLXNA1 variants have been reported. Dworschak et al. (32) described four families with biallelic and three with monoallelic *de novo* variants. Shared core features among these cases include global developmental delay, brain anomalies, and eye malformations, with seizures being more common in individuals with monoallelic mutations. Morpholino knockdown of zebrafish homologs (plxna1a and plxna1b) results in central nervous system and ocular anomalies, mirroring patient presentations.

Notably, a left CDH was observed in the fetus—a phenotype not currently associated with PLXNA1 variants. CDH is a rare developmental defect of the diaphragm with an estimated livebirth incidence of 1/2,500–1/3,000 (33). Approximately 30%−50% of CDH cases are complex, often co-occurring with structural, chromosomal, and/or monogenic disorders (34). A meta-analysis of 5,927 CDH cases reported a 10% rate of genetic abnormalities, including 7.3% chromosomal anomalies (e.g., T18, T13, T21) and 2.7% monogenic syndromes (e.g., Fryns syndrome, Cornelia de Lange syndrome) (35). In this case, CMA ruled out chromosomal aneuploidies and pathogenic/likely pathogenic CNVs >100 kb. WGS also excluded variants in known CDH-associated monogenic syndromes. Therefore, the CDH in this case may represent a novel phenotypic expansion of PLXNA1-related disorders, or it could result from an unrecognized recessive variant in another CDH-associated gene, or from a homozygous variant in an as-yet-unknown gene on chromosome 3 contributing to the overall clinical phenotype.

## 5 Limitations of the study

This study has several limitations that must be acknowledged. First, the diagnosis of CPM remains presumptive due to the unavailability of placental tissue for direct molecular analysis. Second, the assessment of the functional impact of the PLXNA1 variant lacked validation through biochemical or cellular experiments, which constitutes a major limitation of this work.

## 6 Conclusions

This study reports a case of mixed maternal UPD3, suggesting that uniparental disomy may be a potential mechanism for unmasking recessive disorders. These findings indicate that comprehensive genomic analysis is crucial for achieving accurate diagnosis and genetic counseling when non-invasive prenatal testing and chromosomal microarray analysis yield discordant results.

## Data Availability

The datasets presented in this study can be found in online repositories. The names of the repository/repositories and accession number(s) can be found in the article/supplementary material.
